# An Emblem of Decay

**Published:** 1921-02-05

**Authors:** 


### "An Emblem of Decay."

Calling attention in a recent issue to the possibilities of the National Training-school for District Midwives at Woolwich, which is now in course of erection, *Maternity and Child Welfare* points the moral by giving instances of deaths which occur among infants through gross ignorance and an attitude of passive acquiescence on the part of the ignorant mother. That attitude of acquiescence, the writer points out, is a condition of things which we know to be unnecessary and a factor with which all welfare workers have to reckon.

Dr. Foote makes the striking suggestion that the nursing-bottle may be taken to symbolise national decay, pointing out that, as surely as a people has become wealthy and degenerate, the tendency to abandon breast-feeding has followed. There is much to hope for, in England at any rate, from the spread of knowledge by doctors, midwives, and health visitors and the growing "sense" of hygiene in the body politic. Women are certainly beginning to realise first that they need not "reckon" to lose babies, and, next, that "the preservation of babies in life and health during early months may be assured by avoiding that emblem of national downfall, the feeding-bottle." We hope the time will come when it will be a matter of pride for a mother to say that her child is breast-fed, and a matter of shame, except from necessity, to say that her child is fed by the bottle.

